# Epiligament Tissue of the Medial Collateral Ligament in Rat Knee Joint: Ultrastructural Study

**DOI:** 10.7759/cureus.3812

**Published:** 2019-01-02

**Authors:** Georgi P Georgiev, Alexandar Iliev, Georgi Kotov, Violeta K Nedialkova, Vidin Kirkov, Boycho Landzhov

**Affiliations:** 1 Orthopaedics and Traumatology, Queen Giovanna Hospital, Sofia, BGR; 2 Pathology, Medical University of Sofia, Sofia, BGR; 3 Orthopaedics and Traumatology, Medical University of Sofia, Sofia, BGR; 4 Pathology, Medical University Sofia, Sofia, BGR; 5 Orthopaedics, Medical University of Sofia, Sofia, BGR

**Keywords:** epiligament, medial collateral ligament, rat

## Abstract

Introduction

Recent studies stressed the importance of the epiligament in ligament nutrition and healing. While ligaments of the knee joint have been the subject of extensive research, the epiligament of the medial collateral ligament has received only limited attention. The aim of our study was to present the ultrastructural morphological features of the epiligament of the medial collateral ligament in a rat knee joint.

Materials and methods

For the present study, we used eight eight-month-old male Wistar rats. A transmission electron microscopic study of the epiligament was conducted according to standard protocol.

Results

In the epiligament, we described the presence of fibroblasts with the typical features of protein-synthesizing cells, as well as fibrocytes and adipocytes. We noted an abundance of blood vessels and nerve elements. Collagen fibers were organized in multidirectional bundles.

Conclusions

Our findings confirm that the cells and structures of the epiligament play an important role in the nutrition and healing of the medial collateral ligament.

## Introduction

The surface layers of connective tissue associated with bone, cartilage, striated muscle, nerve, and tendon are termed periosteum, perichondrium, epimysium, epineurium, and epitenon, respectively [1]. In 1990, Bray et al. defined, for the first time, the term “epiligament” (EL) as a “surrounding adherent connective tissue removed simultaneously with the ligament but which was grossly distinguishable from ligament tissue proper” [2]. Recent studies stressed the importance of the EL in ligament nutrition and healing [3-15].

Ligaments of the knee joint have been the subject of extensive research (by transmission electron microscopy) while the EL of the medial collateral ligament (MCL) received only limited attention. The latter was examined in current literature by several studies describing the ultrastructural morphology of the EL in mature animals or comparing mature and immature animals [3-5].

The aim of our study was to present the ultrastructural morphological features of the EL of the MCL in a rat knee joint.

## Materials and methods

For the present study, we used eight eight-month-old male Wistar rats. All animal procedures conformed to the guidelines in Directive 2010/63/EU of the European Parliament concerning the protection of animals used for scientific purposes. All experiments were conducted with the approval of the University Committee on Animal Resources (No. 4866). All animals received humane care in compliance with the ‘Principles of laboratory animal care’ formulated by the National Society for Medical Research and the ‘Guide for the care and use of laboratory animals’ prepared by the National Institute of Health (NIH publication No. 86–23, revised 1996). The study was approved by the Medical Legal Office, the Local Ethics Committee, and the Institutional Review Board. The animals were sacrificed after an overdose of ether. The MCL and surrounding EL were dissected precisely, and then the pieces were fixed in 3% glutaraldehyde (Merck Catalogue No. 354400, Merck KGgA, Darmstadt, Germany) for two hours. After fixation, the tissues were rinsed several times with 0.1% phosphate buffer (Merck Catalogue No. 1465920006) to remove the fixative solution and were incubated in 1% osmium tetroxide (Merck Catalogue No. 1245050500) for two hours. Then, the pieces were dehydrated in EtOH (50, 70, 95, 100%) (Merck Catalogue No. 1009835000) and treated for 30 minutes with propylene oxide (Merck Catalogue No. 807027). The pieces were embedded in Durcupan (Fluka, Buchs, Switzerland). Afterward, all slices were processed with a dissectional microscope and cut by an ultramicrotome (LKB, Stockholm-Bromma, Sweden). The EL regions were identified on semi-thin sections (1 µm thick). Ultrathin sections (60 nm thick) were taken only from the MCL EL and then transferred to copper grids (300 mesh) and contrasted with 2.5% uranyl acetate (Electron Microscopy Sciences Catalogue No. 102092-284, Electron Microscopy Sciences, Hatfield, Pennsylvania, United States), lead nitrate (Merck Catalogue No. 1073980100), and sodium citrate (Merck Catalogue No. 1110371000). The samples were observed under a transmission electron microscope Hitachi model H-500 (Hitachi, Ltd., Tokyo, Japan), using an acceleration voltage of 100 kV.

## Results

The EL tissue of the MCL consists of fibroblasts, fibrocytes, fat cells, and neurovascular bundles, as well as single blood vessels. Their ultrastructural characteristics are presented below.

**Figure 1:**
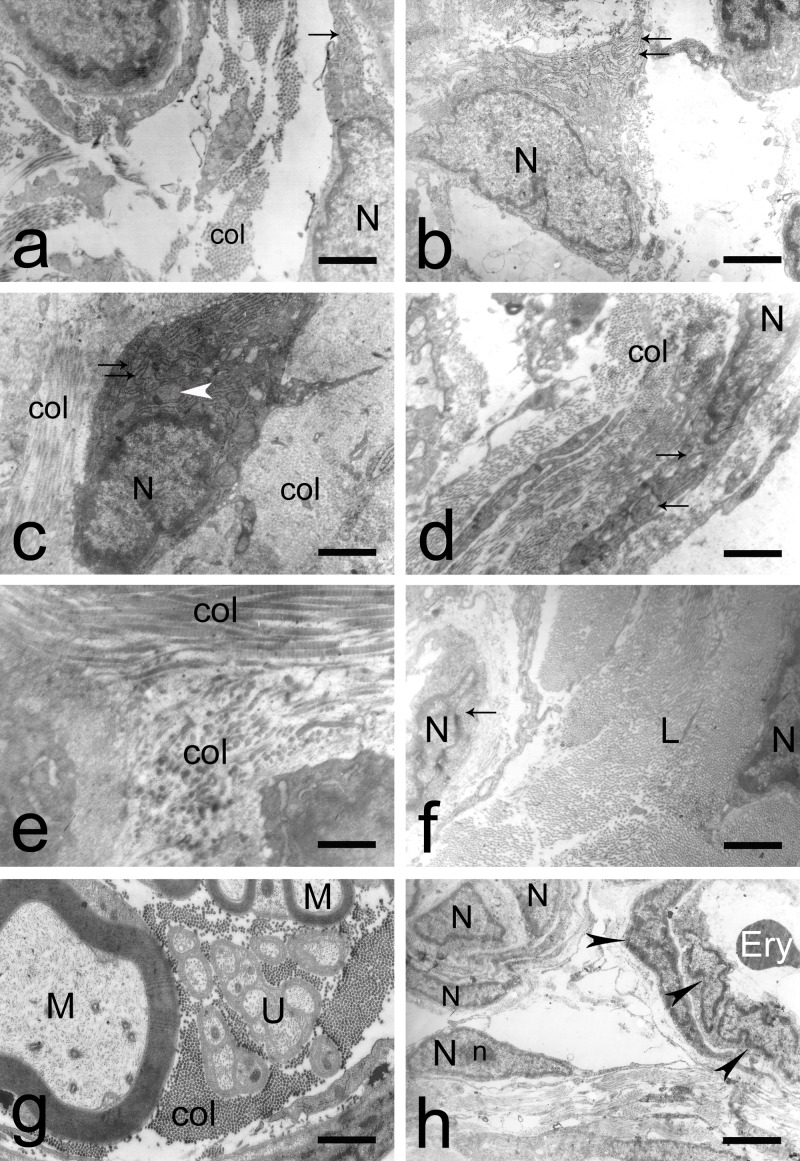
Normal morphology of the external surface of the epiligament of the medial collateral ligament a) Electron micrograph of fibroblasts in the intercellular matrix with a large nucleus (N), well-presented rough endoplasmic reticulum (arrow), and collagen fibers (col); Scale bar - 1.5 µm; b) Electron micrograph of fibroblast in the intercellular matrix with a large nucleus (N) and well-presented rough endoplasmic reticulum (arrows); Scale bar - 2.3 µm; c) Electron micrograph of fibroblast in the extracellular matrix with a large nucleus, prominent rough endoplasmic reticulum (arrows), spherical mitochondria (white arrowhead), and collagen fibers (col); Scale bar - 1.1 µm; d) Electron micrograph of fibroblasts in the intercellular matrix with a large nucleus (N), well-presented rough endoplasmic reticulum (arrows), and collagen fibers (col); Scale bar - 2.3 µm; e) Electron micrograph of multidirectional collagen fibers (col) in the epiligament; Scale bar - 0.8 µm; f) Epiligament with fibroblast in the intercellular matrix with a large nucleus (N) and well-presented rough endoplasmic reticulum (arrow). The border of the epiligament prolongs into the endoligament towards the ligament (L) proper. Fibroblast in the ligament with a well-shaped nucleus (N) is also seen; Scale bar - 2.3 µm; g) Electron micrograph of myelinated (M) and unmyelinated fibers (U) in the epiligament. Col - collagen fibers; Scale bar - 1.1 µm; h) Epiligament with fibroblast with triangular shape and spindle-shaped fibrocytes in the intercellular matrix with large nucleus (N), blood vessel with erythrocytes (Ery) and pericytes (arrowheads) around the blood vessel wall. n - nucleolus; Scale bar - 1.5 µm.

The fibroblasts were large and well formed. They displayed a very delicate chromatin structure and a prominent nucleolus. Their cytoplasm contained a considerable number of free ribosomes, polysomes, well-presented rough endoplasmic reticulum, poorly developed Golgi apparatus, spherical mitochondria, and single lysosomes. Spindle-shaped fibrocytes with similar nuclei and rough endoplasmic reticulum were reported too. The third cell type was presented by unilocular spherical and polyhedral adipose cells, which were found closely packed with one another. The adipocytes had large vacuoles and eccentric, flat nuclei, surrounded by a basal lamina (Figures 1a-1d). In the intercellular space, we discovered collagen fibers with multiple orientations (Figure 1e). In the same layer, myelinated and unmyelinated nerve fibers formed nerve trunks. The external fibrous coat of the trunk (epineurium) filled the space between the bundles of nerve fibers, each surrounded by perineurium. Schwann cells were also detected. The blood vessels had thin walls and their intima consisted of layers of endothelial cells, with a number of pericytes around them (Figures 1f-1h).

## Discussion

According to Chowdhury et al., who described the external surface of the MCL in rabbits, there were two types of cells – spinous-shaped fibroblasts and fat cells, which differed from ligament cells [1]. These authors did not present the ultrastructural findings in detailed description. On the contrary, fibroblasts in the EL of MCL were of various types: spinous-shaped, spindle-shaped, elongated, and with irregular form [3,5,7]. The data from the present study gave a detailed examination of the morphological changes in the midsubstance of the EL of MCL, fibroblasts, fibrocytes, fat cells, and neurovascular bundles in particular. Similar ultrastructural characteristics were discussed by Georgiev et al. [12]. Their study described the special characteristics of fibroblasts, supporting the hypothesis of their importance in the nutrition and healing of the MCL, along with the abundant blood vessels.

Chowdhury et al. speculated that the spinous-shaped cells are primarily responsible for the synthesis of collagen fibers [1]. Our observations supported those earlier reports. Fat cells in the EL appear to be typical adipocytes that form white adipose tissue [16]. These cells metabolize and store lipids and may function as a packing material that could confer the distinct properties of the EL [1]. It is well-known that both small and large collagen fibers in the ligament are aligned parallel to its long axis and form fascicles separated by the transverse sections of septae while the whole structure is enveloped in thin connective tissue called endoligament [16]. In contrast, our research showed uniform small collagen fibers in the EL organized in bundles with multiple orientations and that the fibers of the EL of MCL continued as the endoligament. The EL layer appeared to contain a relative abundance of blood vessels and nerve elements compared to the ligament tissue [5]. The blood vessels in the EL were randomly dispersed in a loose connective tissue matrix while the nerve bundles often accompanied them, but apparently not all blood vessels formed part of a neurovascular bundle [2,5]. We found that the EL of the MCL had both myelinated and unmyelinated nerve fibers, which occurred frequently in contrast to the ligament [17]. The axonal density of nerve fiber distribution is the greatest in the fibular collateral ligament in the knee joint of rats as compared to the other ligaments [18]. On the contrary, nerve types and their endings found in the MCL during an examination by McDougall et al. appeared to have a similar innervation pattern as the medial collateral ligament [19].

## Conclusions

Different types of cells and cell complexes (fibroblasts, fibrocytes, and fat cells) can be found in the EL of the MCL. Fibroblasts in the EL may be involved in processes such as differentiation, phagocytosis, and collagen synthesis due to their ultrastructural characteristics. Collagen fibers in the midsubstance of the external surface of EL in the MCL have uniformly small diameters and are organized in bundles with varying orientations, opposed to collagen fibers in the ligament. These differences in orientation suggest that the EL may respond to tension differently in different directions having better mobility than the ligament. The EL contains a multitude of blood vessels and nerve fibers (the latter can be both myelinated and unmyelinated), as opposed to the ligament substance.
