# Correction: Epiligament Tissue of the Medial Collateral Ligament in Rat Knee Joint: Ultrastructural Study

**DOI:** 10.7759/cureus.c19

**Published:** 2019-03-07

**Authors:** Georgi P Georgiev, Alexandar Iliev, Georgi Kotov, Violeta K Nedialkova, Vidin Kirkov, Boycho Landzhov

**Affiliations:** 1 Orthopaedics and Traumatology, Queen Giovanna Hospital, Sofia, BGR; 2 Department of Anatomy, Histology and Embryology, Medical University of Sofia, Sofia, BGR; 3 Student, Faculty of Medicine, Medical University of Sofia, Sofia, BGR

The author affiliations were incorrect upon publication. The article has been amended to include the correct affiliations:

Georgi P. Georgiev (1), Alexandar Iliev (2), Georgi Kotov (2), Violeta K. Nedialkova (3), Vidin Kirkov (3), Boycho Landzhov (2)

1. Orthopaedics and Traumatology, Queen Giovanna Hospital, Sofia, BGR
2. Department of Anatomy, Histology and Embryology, Medical University of Sofia, Sofia, BGR
3. Student, Faculty of Medicine, Medical University of Sofia, Sofia, BGR

